# The impact of the multidisciplinary Endocarditis Team on the management of infective endocarditis

**DOI:** 10.1007/s12471-022-01707-6

**Published:** 2022-07-04

**Authors:** A. R. Wahadat, W. Tanis, T. W. Galema, L. E. Swart, W. J. van Leeuwen, N. J. Verkaik, C. A. M. Schurink, B. van Dalen, S. Zoet-Nugteren, C. Gurlek, R. P. J. Budde, J. W. Roos-Hesselink

**Affiliations:** 1 Department of Cardiology, Erasmus Medical Centre, Rotterdam, The Netherlands; 2 Department of Radiology and Nuclear Medicine, Erasmus Medical Centre, Rotterdam, The Netherlands; 3 Department of Cardiology, Haga Teaching Hospital, The Hague, The Netherlands; 4 Department of Cardiothoracic Surgery, Erasmus Medical Centre, Rotterdam, The Netherlands; 5 Department of Medical Microbiology and Infectious Diseases, Erasmus Medical Centre, Rotterdam, The Netherlands; 6 Department of Internal Medicine, Erasmus Medical Centre, Rotterdam, The Netherlands; 7 Department of Cardiology, Franciscus Gasthuis & Vlietland Hospital, Rotterdam, The Netherlands; 8 Department of Cardiology, Ikazia Hospital, Rotterdam, The Netherlands; 9 Department of Cardiology, Admiral de Ruyter Hospital, Goes, The Netherlands

**Keywords:** Infective endocarditis, Infection, Multidisciplinary care

## Abstract

**Background:**

In their latest guidelines for infective endocarditis (IE) (2015), the European Society of Cardiology (ESC) introduced the implementation of the Endocarditis Team (ET) to facilitate the management of IE. This study presents our experiences and the diagnostic and therapeutic impact of the ET on the management of IE.

**Methods:**

From 2016–2020, data of all patients with suspected IE referred to the ET were prospectively collected. The final diagnosis was defined by the ET as either rejected, possible or definite IE. Diagnostic impact was scored as any change in initial diagnosis, the frequency of additional diagnostic tests advised by the ET and any change in diagnosis after these tests. Therapeutic impact was scored as any change in antibiotic therapy or change from conservative to invasive therapy or vice versa.

**Results:**

A total of 321 patients (median age 67 [55–77] years, 71% male) were enrolled. The final diagnosis was rejected IE in 47 (15%), possible IE in 34 (11%) and definite IE in 240 (75%) patients. A change of initial diagnosis was seen in 53/321(17%) patients. Additional microbiological tests were advised in 69/321 (21%) patients, and additional imaging tests in 136/321 (42%) patients, which resulted in subsequent change in diagnosis in 23/321 (7%) patients. Any change in antibiotic treatment was advised in 135/321 (42%) patients, and change from initial conservative to additional surgical treatment in 15/321 (5%) patients.

**Conclusion:**

The ET had a clear impact on the therapeutic policy for patients with suspected IE and is useful in the management of this life-threatening disease. Broad implementation is warranted.

**Supplementary Information:**

The online version of this article (10.1007/s12471-022-01707-6) contains supplementary material, which is available to authorized users.

## What’s new?

- This manuscript provides data of 4 years of experience with an Endocarditis Team from a prospective registry in a tertiary reference centre
- Endocarditis Team recommendations can result in a 17% change in diagnosis
- Endocarditis Team recommendations can result in a 42% change in antibiotic treatment
- Endocarditis Team recommendations can result in a 5% change from conservative to invasive treatment

## Introduction

Despite ongoing improvements in diagnosis and treatment, infective endocarditis (IE) remains associated with high morbidity and mortality [1–5]. Due to the intricacy of the disease and its treatment, management by a single practitioner will be suboptimal [6].

A multidisciplinary approach of IE can help decrease its mortality [7, 8] and, therefore, the European Society of Cardiology (ESC) advocates the instalment of an “Endocarditis Team” (ET) in each reference centre [1]. After this recommendation by the ESC, other studies have shown the importance of an ET by reporting mortality reduction after its implementation [9, 10]. However, there are no data about how exactly an ET influences the management of IE and, if so, to what extent it changes the initial diagnostic and therapeutic policy.

This paper presents our experiences of our ET in the first 4 years after initiation to provide detailed information about its impact on the management of IE.

## Methods

### Setting up the Endocarditis Team

In 2016, an ET was instated at our institution, including at least a cardiologist with particular expertise in echocardiography, a cardiothoracic surgeon, a medical microbiologist or infectious disease specialist, a cardiovascular radiologist, a nuclear medicine physician and a coordinator. All regional cardiologists were informed about the ET by letter and via a newsletter emailed to the regional society of cardiologists, both containing information on how to refer patients to our team. Initially, the referring cardiologist sought contact by phone or email, followed by written information about the case in the form of a letter. In addition, all available images of the diagnostic tests such as transthoracic and transoesophageal echocardiography (TTE/TOE), cardiac computed tomography angiography (CTA) and positron emission tomography with computed tomography (PET/CT) were sent to the ET.

Regular sessions were planned biweekly and additional ad hoc sessions were planned in urgent cases.

## Data registration

All patients discussed in the ET were prospectively anonymously entered into a database. The final diagnosis was decided by the ET to either be rejected, possible or definite IE at the end of all ET meetings. Patients with a final diagnosis of definite IE were divided into three groups (group 1: native; group 2: prosthetic valves/prostheses; group 3: cardiac devices). Patients with both native and prosthetic valve infection or both cardiac device and prosthetic valve infection, were included in group 2. Patients with both native valve and cardiac device infection were included in group 3. Information on patient follow-up was derived from the electronic patient records. Follow-up time was defined as the period between the discussion date until the date of the last notation in the clinical records. Relapse was defined as recurrence of IE by the same micro-organism and within 6 months after the first episode. Re-infection was defined as recurrence after 6 months. Data about mortality were derived from the database of Statistics Netherlands (*Centraal Bureau voor de Statistiek*—CBS). The need for informed consent was waived by the local Medical Research Ethics Committee.

## Diagnostic and therapeutic impact

Diagnostic impact was scored as the number of reclassified patients (rejected, possible and definite IE). If a diagnosis was not provided beforehand by the referring physician, the final diagnosis by the ET was not scored as reclassification. However, if the additional diagnostic tests advised by the ET did change the initial diagnosis by the ET in the first meeting, this was scored as reclassification.

Therapeutic impact was scored as the combination of any change in antibiotic therapy and/or change in either conservative or invasive treatment resulting from additional diagnostic tests advised by, or decisions made by, the ET.

## Statistics

We used descriptive statistics for the analyses of the main outcomes. Categorical variables were reported as numbers and percentages, whereas continuous variables were reported as mean ± standard deviation (SD) or medians and interquartile ranges (IQR). Non-parametric statistical analyses (Mann-Whitney U test) were performed for the comparison of two continuous variables. The Kruskal-Wallis test was used for comparison of > 2 continuous variables, and for categorical variables the chi-squared (*X*^*2*^) test was performed to determine differences between groups. Kaplan-Meier curve plots for time to all-cause mortality and time to rate of relapse/re-infection were made, and a log-rank test was performed to demonstrate the difference between groups for either survival or relapse/re-infection. We used a significance level of *p* = 0.05.

## Results

Between January 2016 and January 2020, 321 unique patients (median [IQR] age 67 years [55–77]; male: *n* = 228, 71%) with suspected IE from 10 different medical centres (95/321, 29.5% from our own centre) were referred to our ET. None of the other participating hospitals had implemented their own ET. In all cases, the reason for referring the patients was simply to adhere to the ESC guideline recommendations to refer all patients with suspected IE (including uncomplicated cases) to the ET. An overview of the baseline characteristics is presented in Tab. 1.

**Table 1:** Patient demographics and characteristics

	Total (*n* = 321)	Rejected IE (*n* = 47)	Possible IE (*n* = 34)	Definite IE (*n* = 240)			*P*-value ^a^
				*Native valve* *(n* *=* *125)*	*Prosthesis* *(n* *=* *96)*	*Cardiac device* *(n* *=* *19)*	
Gender = male, *n* (%)	228 (71)	31 (66)	25 (74)	172 (72)			0.69 0.15
				* 85 (68)*	* 70 (73)*	*17 (89)*	
Median age (years) [IQR]	67 [55–77]	66 [57–75]	64 [55–74]	68 [54–77]			0.69 0.34
				* 66 [54–76]*	* 71 [55–78]*	*66 [61–75]*	
Previous IE	21 (7)	4 (9)	4 (12)	13(5)			0.33 0.02
				* 3 (2)*	* 10 (11)*	* 0 (0)*	
CHD	34 (11)	3 (6)	5 (15)	26 (11)			0.45 0.001
				* 6 (5)*	* 19 (20)*	* 1 (5)*	
History of heart failure	36 (11)	9 (20)	7 (21)	20 (8)			0.02 0.01
				* 8 (6)*	* 7 (7)*	* 5 (26)*	
Valve disease	172 (54)	25 (53)	18 (53)	129 (54)			0.99 < 0.001
				* 39 (31)*	* 84 (88)*	* 5 (26)*	
Cardiac device	70 (22)	16 (34)	14 (41)	40 (17)			0.001 < 0.001
				* 8 (6)*	* 13 (14)*	*19 (100)*	
DM	59 (18)	7 (15)	8 (24)	44 (18)			0.59 0.60
				* 20 (16)*	* 20 (21)*	* 4 (21)*	
Hypertension	96 (30)	9 (19)	11 (32)	76 (32)			0.19 0.17
				* 45 (36)*	* 28 (29)*	* 3 (16)*	

*IE* infective endocarditis, *CHD* congenital heart disease, *DM* diabetes mellitus

^a^ *P*-value for the difference between rejected, possible and definite IE on the top of each box. The bottom *p*-value is the difference between native, prosthetic and cardiac device IE for patients with the final diagnosis of definite IE

Positive blood cultures were seen in 276/321 (86%) patients, with *Staphylococcus aureus *(*S. aureus*) as the most common pathogen (*n* = 76, 24%). An overview of the microbiological test results are presented in Table S1 in the Electronic Supplementary Material. Echocardiography was positive for IE in 206/321 (64%) cases. The positive echocardiographic findings were vegetations (158/206, 77%), abscess (6/206, 3%), mycotic aneurysms/paravalvular leaks (9/206, 4%), new valvular insufficiency/stenosis (8/206, 4%) and combination of vegetations/abscess/paravalvular and/or valvular insufficiency (25/206, 12%). PET/CT was performed in 152/321 (47%) patients and was positive in 80/152 (53%) patients. CTA was performed in 84/321 (26%) patients and was positive in 63/84 (75%) patients. An overview of all diagnostic imaging results is presented in Tab. 2.

**Table 2:** Diagnostic imaging performed

Imaging diagnostics	Total (*n* = 321)	Rejected IE (*n* = 47)	Possible IE (*n* = 34)	Definite IE (*n* = 240)			*P*-value ^a^
				*Native valve* *(n* *=* *125)*	*Prosthesis* *(n* *=* *96)*	*Devices* *(n* *=* *19)*	
Positive imaging *n* (%)	252 (79)	14(30)	16(47)	222(93)			< 0.001 0.27
				*118(94)*	* 88(92)*	*16(84)*	
TTE/ TEE performed *n* (%)	321 (100)	47(100)	34(100)	240(100)			NA NA
				*125(100)*	* 96(100)*	*19(100)*	
Positive TTE/TEE *n* (%)	206 (64)	8(17)	8(24)	190(79)			< 0.001 < 0.001
				*117(94)*	* 60(63)*	*13(68)*	
PET/CT performed *n* (%)	152 (47)	23(49)	18(53)	111(46)			0.74 < 0.001
				* 32(26)*	* 70(73)*	* 9(47)*	
Positive PET/CT *n* (%)	80 (25)	3(6)	7(21)	70 (29)			< 0.001 < 0.001
				* 8(6)*	* 59(61)*	* 3(16)*	
CTA performed *n* (%)	84 (26)	12(26)	10(29)	62(26)			0.90 < 0.001
				* 13(10)*	* 47(49)*	* 2(11)*	
Positive CTA *n* (%)	63 (20)	5(11)	4(12)	54(23)			< 0.001 0.23
				* 13(10)*	* 39(41)*	* 2(11)*	

*IE* infective endocarditis, *TTE* transthoracic echocardiogram, *TEE* transoesophageal echocardiogram, *PET/CT* positron emission tomography/computed tomography,* CTA* cardiac computed tomography angiography

^a^ *P*-value for the difference between rejected-, possible- and definite IE on the top of each box. The bottom *p*-value is the difference between native, prosthetic and cardiac device IE for patients with the final diagnosis of definite IE

**Fig. 1:**
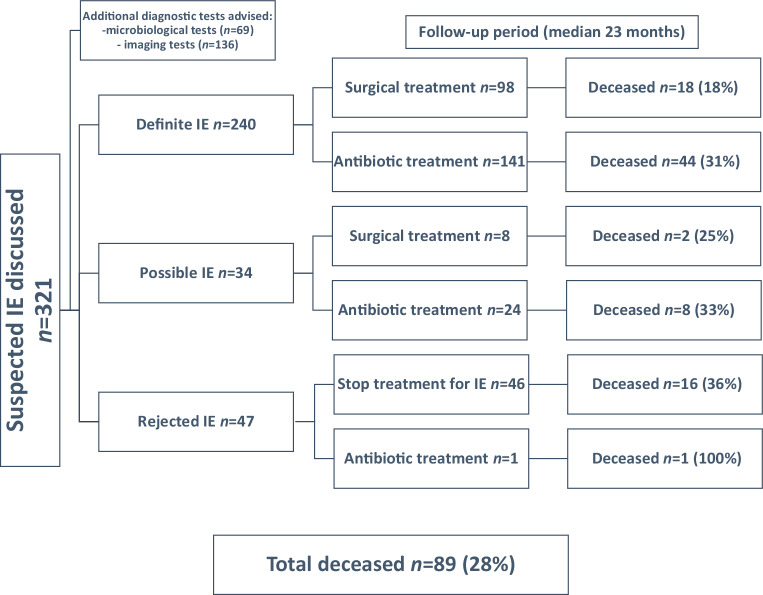
Overview of the results of 321 patients with suspected IE discussed in the Endocarditis Team. *IE* infective endocarditis

The final diagnosis by the ET was 47 (15%) rejected, 34 (11%) possible and 240 (75%) definite IE with 125/240 (52%) native valve IE, 96/240 (40%) prosthetic valve IE and 19/240 (8%) cardiac device-related IE. In 7/240 (3%) patients, both a native and prosthetic valve (*n* = 6) or prosthetic valve and cardiac device (*n* = 1) were involved. An overview of the results of all discussed patients is presented in Fig. 1.

## Diagnostic and therapeutic impact

The ET advised additional microbiologic tests and imaging tests in 69/321 (21%) and 136/321 (42%) cases respectively. The reason for additional microbiologic tests were either an initial negative test or only one set of positive microbiologic tests in patients with high suspicion of IE. The results of these tests were negative in 21/69 (30%) and positive in 48/69 (70%) patients. A change in diagnosis was observed in 53/321 (17%) patients, out of which 39/53 (74%) with reclassification from possible to rejected IE, 4/53 (8%) from rejected to possible IE and 10/53 (19%) from possible to definite IE. Reclassification was the result of the advised additional diagnostic tests in 23/53 (43%) patients. In all other cases reclassification resulted from revision of patient data and the clinical images that were provided. Due to the advised additional diagnostic tests, there was a change in treatment in 31/321 (10%) patients. In 16/321 (5%) patients, these changes were solely in antibiotic treatment and in 15/321 (5%) the change was from a proposed conservative to a surgical management. Details per group are demonstrated in Tab. 3.

**Table 3:** Additional diagnostic tests advised by the Endocarditis Team

Additional diagnostic tests	Total (*n* = 321)	Rejected IE (*n* = 47)	Possible IE (*n* = 34)	Definite IE (*n* = 240)			*P*-value ^a^
				*Native valve* *(n* *=* *125)*	*Prosthesis* *(n* *=* *96)*	*Devices* *(n* *=* *19)*	
Microbiological tests *n* (%)	69 (21)	15 (32)	10 (29)	44 (18)			0.06 0.60
				* 26(21)*	*15(16)*	*3(16)*	
Imaging tests *n* (%)	136(42)	17(36)	17(50)	102(43)			0.46 0.03
				* 49(39)*	*49(51)*	*4(21)*	
Diagnostic change due to additional diagnostic tests *n* (%)	23(7)	8(17)	2(6)	13(5)			0.02 0.01
				* 2(2)*	*11(12)*	*0(0)*	
Therapeutic change due to additional diagnostic tests *n* (%)	31(10)	5(11)	2(6)	24(10)			0.14 0.15
				* 10(8)*	*14(15)*	*0(0)*	
Change to invasive treatment due to additional microbiological tests *n* (%)	2(1)	0(0)	0(0)	2*(1)*			0.18 0.59
				* 1(1)*	* 1(0)*	*0(0)*	
Change to invasive treatment due to additional imaging tests *n* (%)	13(4)	0(0)	1(3)	12(5)			0.05 0.35
				* 5(4)*	* 7(7)*	*0(0)*	

*IE* infective endocarditis

^a^ *P*-value for the difference between rejected, possible and definite IE on the top of each box. The bottom *p*-value is the difference between native, prosthetic and cardiac device IE for patients with the final diagnosis of definite IE

## Conservative and surgical treatment

An overview of the therapeutic policy for the total study population is presented in Table S2 in the Electronic Supplementary Material. Antibiotic treatment alone (without surgical intervention) was used in 166/321 (52%) (141 definite IE) patients. Surgical intervention (alongside antibiotics) was needed in 107/321 (33%) (98 definite IE) patients. Device extraction was performed in 16/20 (80%) patients with definite cardiac device-related IE. No treatment advice was given to 48/321 (15%) out of which 47 patients with rejected IE and 1 patient with possible IE who was already receiving antibiotics for another infection and who showed no signs of active endocarditis at the moment of discussion.

## Follow-up

Two patients were lost to follow-up due to moving to another country shortly after hospitalisation.

**Fig. 2:**
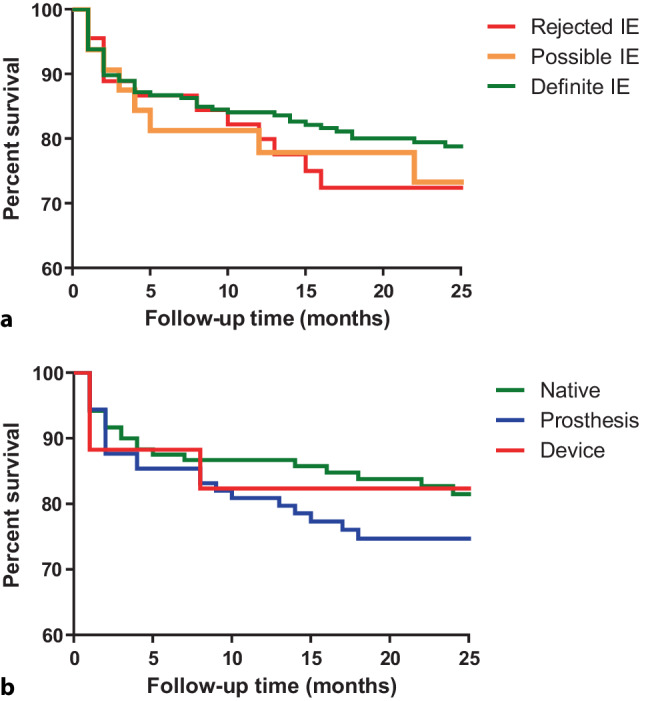
The survival curve of all discussed patients per group (**a**) and the patients with definite IE per category (**b**). *IE* infective endocarditis

During a median follow-up period of 23 [12–38] months, the mortality rate for patients with a final diagnosis of rejected, possible and definite IE was 17/47 (36%), 10/34 (29%) and 62/240 (26%) respectively (*p* = 0.08). For native valve IE, prosthesis IE and cardiac device-related IE the mortality rate was 25/125 (20%), 32/96 (33%) and 5/19 (26%) respectively (*p* = 0.78). The cause of death could not always be derived from the follow-up data. Fig. 2 demonstrates the survival curves of each group.

The 30-day mortality rate of patients who underwent surgical treatment was 8/107 (7%); 1 with elective surgery, 2 with device extraction and 5 with urgent surgery. The 30-day mortality rate of patients with definite cardiac device-related IE without device extraction was one in four (25%).

**Fig. 3:**
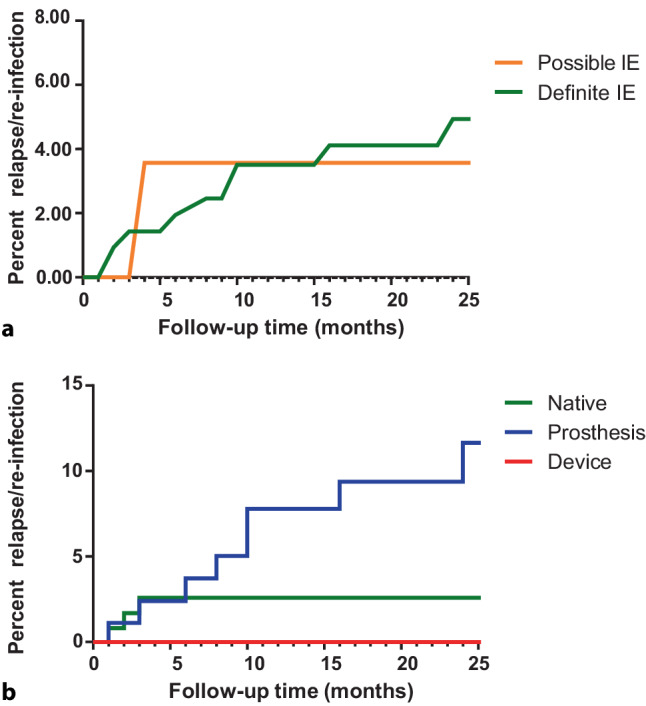
The incidence curve for relapse/re-infection of all patients with possible and definite IE (**a**) and patients with definite IE per category (**b**). *IE* infective carditis

The rate of relapse/re-infection during the follow-up period was 10/240 (4.2%) for definite IE; 4 (1.7%) patients with relapse and 6 (2.5%) patients with re-infection. For native valve IE, prosthesis IE and cardiac device-related IE, the rates of relapse/re-infection were 2/125 (1.6%), 8/96 (8%) and 0/19 (0%) respectively (*p* = 0.03). The incidence rate for relapse/re-infection is shown in Fig. 3.

## Discussion

The present study shows that the ET provided a 17% change in diagnosis, 42% change in antibiotic treatment and 5% change from conservative to invasive treatment. This implies that a multidisciplinary approach results in a difference in policy in about half of the patients.

The difference in advice from the ET relative to the policy of the referring physician could be explained by the level of information available to the physicians of the ET compared with that available to the physicians from the referring hospitals. In some cases, the ET members had less information about the microbiological results, such as missing susceptibility values. In other cases, the ET members had more information than the referring physician, due to the additional diagnostic imaging results that were revised by the ET. Another explanation could be that ET members had more experience in the treatment of specific patients with IE and were thus able to provide an expert opinion.

Additional advised diagnostic tests changed the diagnosis in only 23/321 (7%) patients, and were primarily advised to rule out complications of IE. Although this could be seen as a defensive strategy, it was according to the ESC guidelines and it did lead to a change in therapy in 31/321 (10%) patients, that otherwise could have suffered from severe consequences.

The mortality rate in our study (28%) did not differ between rejected, possible and definite IE, nor did it differ between the native, prosthesis and cardiac device-related IE groups (Fig. 2). Compared with other studies that report an in-hospital mortality of 17% in the first 2 months after diagnosis and 27% in the first 6 months after diagnosis [5, 11], the mortality rate reported in our study after a median follow-up time of 23 months can be interpreted as slightly better. The mortality rate in our study can be partially explained by the possible referral bias; complicated cases are more likely to be referred than uncomplicated cases, and very severe cases with no hope for response to treatment can be left untreated and not be referred. The exact incidence number of patients with IE in the Netherlands is unknown. However, it could be presumed that this number is close to 45 patients per one million inhabitants per year, which has also been described in other European countries [1]. The region of our hospital has 1.58 million inhabitants which equals an incidence of 284 patients per 4 years. This number is slightly higher than the possible/definite IE cases in our study, which indicates that we have not included all patients from the region.

Multiple challenges can be encountered in the process of setting up an ET, such as determining and inviting the required specialists who should attend the meeting. Nowadays, imaging techniques such as PET/CT and CTA are advised to be used more often for the diagnosis of IE [1]. In our opinion, the presence of a cardiac radiologist or cardiac imaging specialist during ET discussions is important, since the recently introduced imaging techniques can sometimes be difficult to interpret in light of their limitations and technical aspects that need to be considered. Other challenging aspects of setting up an ET may be the timing and location of the meetings, the preparation of the cases and collecting all information necessary for a meaningful discussion. These challenges could be met by having a coordinator who has an overview of the cases that need to be discussed and who plays a key role in the management of these tasks. Furthermore, with digital communication solutions becoming more and more generally available in hospitals since the Covid-19 pandemic, we also see opportunities for more interactive meetings with attending physicians from referring centres, which would not only provide more insight in the ET’s considerations, but could also serve an important educational purpose [12].

Our study has some limitations, such as the incapability of providing a true population-based sample of patients with IE. We relied on other referring centres for the number of patients discussed in our Endocarditis Team. Furthermore, we also relied on the referring centres to provide us with a complete set of information, which was not always available. Another limitation is that it was not possible to verify whether the provided advice by the ET was adhered to by the referring physician. This may have influenced the follow-up outcomes of both mortality and relapse/re-infection rates.

In conclusion, the ET has a large impact on the therapeutic policy for patients with suspected IE with a substantial change in diagnosis and treatment. Therefore, it should be implemented in all tertiary cardiothoracic centres and be optional for other hospitals.

## Supplementary Information

Table S2 Therapeutic policy for IE

Table S1 Blood culture results
