# Supplementary material for: The impact of the multidisciplinary Endocarditis Team on the management of infective endocarditis
Source: Neth Heart J. 2022 Jul 4;31(1):29–35. doi: 10.1007/s12471-022-01707-6 (PMC9807728; doi:10.1007/s12471-022-01707-6)
Supplement: Supplementary file 1 — Table S2 Therapeutic policy for IE [file 12471_2022_1707_MOESM1_ESM.docx]

**Table S1:** Blood culture results

| **Blood cultures** | **Total (n=321)** | **Rejected IE**  **(n=47)** | **Possible IE (n=34)** | **Definite IE (n=240)** | | | | | **P-value*** |
| --- | --- | --- | --- | --- | --- | --- | --- | --- | --- |
|  |  |  |  | ***Native valve (n= 125)*** | | ***Prosthesis***  ***(n=96)*** | | ***Devices***  ***(n=19)*** |  |
| **Positive BC, n (%)** | 276 (86) | 28(60) | 28(82) | 220 (92) | | | | | <0.001  0.61 |
|  |  |  |  | *116(93)* | | *86(90)* | | *18(95)* |  |
| ***S. aureus* n (%)**** | 77 (24) | 10(21) | 11(32) | 56(23) | | | | | 0.46  0.12 |
|  |  |  |  | *26(21)* | | *22(23)* | | *8(42)* |  |
| ***Other Staphylococci* n (%)** | 24 (7) | 5(11) | 0(0) | 19(8) | | | | | 0.17  0.42 |
|  |  |  |  | *9(7)* | | *7(7)* | | *3(16)* |  |
| ***Viridans group Streptococci* n (%)** | 89(28) | 9(19) | 2(6) | 78(33) | | | | | <0.01  <0.01 |
|  |  |  |  | *57(46)* | | *19(20)* | | *2(11)* |  |
| ***Enterococcus faecalis* n (%)** | 32 (10) | 1(2) | 7(21) | 24(10) | | | | | 0.02  0.51 |
|  |  |  |  | *11(9)* | | *12(13)* | | *1(5)* |  |
| ***Enterococcus faecium* n(%)** | 4(1) | 0(0) | 1(3) | 3(1) | | | | | 0.50  0.82 |
|  |  |  |  | 2(2) | 1(1) | | 0(0) | |  |
| ***Other Streptococci* n (%)** | 18 (6) | 3(6) | 1(3) | 14(6) | | | | | 0.77  0.73 |
|  |  |  |  | *6(5)* | | *7(7)* | | *1(5)* |  |
| ***Cutibacterium acnes* n (%)** | 9 (3) | 0(0) | 1(3) | 8(3) | | | | | 0.45  <0.01 |
|  |  |  |  | *0(0)* | | *8(8)* | | *0(0)* |  |
| ***HACEK* n (%)** | 8 (2) | 0(0) | 0(0) | 8(3) | | | | | 0.25  0.01 |
|  |  |  |  | *2(2)* | | *3(3)* | | *3(16)* |  |
| **Other micro-organisms*** n(%)** | 15 (5) | 0(0) | 5(15) | 10(4) | | | | | 0.01  0.13 |
|  |  |  |  | *3(2)* | | *7(7)* | | *0(0)* |  |

IE: Infective endocarditis

BC: Blood Cultures

HACEK: *Haemophilus, Aggregatibacter*, *Cardiobacterium*, *Eikenella*, *Kingella*.

* P-value for the difference between rejected-, possible- and definite IE on the top of each box. The bottom p-value is the difference between native- prosthetic- and cardiac device IE for patients with the final diagnosis of definite IE.

** One patient in this group had positive blood cultures for Methicillin-resistant *Staphylococcus aureus* (MRSA)

**Other micro-organisms: *Abiotrophia defectiva; Campylobacter fetus; Candida glabrata; Candida parapsilosis, Enterobacter cloacae; Fusobacterium necrophorum; Granulicatella adiacens; Klebsiella pneumonia; Lactobacillus rhamnosus; Lactococcus garvieae*; *Listeria monocytogenes; Moraxella catarrhalis; Morganella morganii*
