# Supplementary material for: The impact of the multidisciplinary Endocarditis Team on the management of infective endocarditis
Source: Neth Heart J. 2022 Jul 4;31(1):29–35. doi: 10.1007/s12471-022-01707-6 (PMC9807728; doi:10.1007/s12471-022-01707-6)
Supplement: Supplementary file 2 — Table S1 Blood culture results [file 12471_2022_1707_MOESM2_ESM.docx]

**Table S2** Therapeutic policy for IE

| **Therapeutic policy** | **Total (*n*=321)** | **Rejected IE**  **(*n*=47)** | **Possible IE (*n*=34)** | **Definite IE (*n*=240)** | | | ***P*-value*** |
| --- | --- | --- | --- | --- | --- | --- | --- |
|  |  |  |  | ***Native valve (n= 125)*** | ***Prosthesis***  ***(n=96)*** | ***Devices***  ***(n=19)*** |  |
| **Antibiotic treatment alone (no intervention) n (%)** | 166(52) | 1(2) | 24(71) | 141(59) | | | <0.01  <0.01 |
|  |  |  |  | *71(57)* | *66(69)* | *4(21)* |  |
| **Surgical Intervention n(%)** | 107(33) | 1(2) | 8(24) | 98(41) | | | <0.001  <0.01 |
|  |  |  |  | *53(42)* | *30(31)* | *15(79)* |  |
| Elective surgery n (%) | 40(12) | 0(0) | 4(12) | 36(15) | | | 0.02  0.03 |
|  |  |  |  | *25(20)* | *11(5)* | *0(0)* |  |
| Urgent/emergency surgery n (%)*** | 50(16) | 1(2)** | 2(6) | 47(20) | | | <0.01  0.07 |
|  |  |  |  | *28(22)* | *19(20)* | *0(0)* |  |
| Device extraction n (%)*** | 18(6) | 0(0) | 2(6) | 16(7) | | | 0.19  <0.001 |
|  |  |  |  | *0(0)* | *1(1)**** | *15(79)* |  |
| **Change from conservative to invasive treatment advised by ET n(%)** | 15(5) | 0(0) | 1(3) | 14(6) | | | 0.2  0.29 |
|  |  |  |  | *6(5)* | *8(8)* | *0(0)* |  |

IE: Infective endocarditis

ET: Endocarditis Team

*P-value for the difference between rejected-, possible- and definite IE on the top of each box. The bottom p-value is the difference between native- prosthetic- and cardiac device IE for patients with the final diagnosis of definite IE.

** One patient with rejected IE underwent an urgent valve surgery due to diagnosis of Libman Sacks endocarditis

***One patient underwent concomitant urgent surgery and device extraction for the diagnosis of both PVE and cardiac device-related IE.
